# Comparative Analysis of Viral Communities in Hospital, University and Urban Wastewater by Shotgun Metagenomic Sequencing

**DOI:** 10.3390/ijms27146430

**Published:** 2026-07-20

**Authors:** Alessandra Nappo, Adeel Mumtaz Abbasi, Giulia Berno, Martina Rueca, Flavia Smoquina, Cesare Ernesto Maria Gruber, Lavinia Fabeni, Pietro Giorgio Spezia, Fabrizio Carletti, Daniele Pietrucci, Maya Petricciuolo, Agnese Carnevali, Nico Sanna, Carmine Talarico, Ermanno Federici, Giovanni Chillemi, Fabrizio Maggi

**Affiliations:** 1 Laboratory of Virology and Laboratories of Biosecurity, National Institute for Infectious Diseases Lazzaro Spallanzani—IRCCS, 00149 Rome, Italy; alessandra.nappo@inmi.it (A.N.); giulia.berno@inmi.it (G.B.); martina.rueca@inmi.it (M.R.); flavia.smoquina@inmi.it (F.S.); cesare.gruber@inmi.it (C.E.M.G.); lavinia.fabeni@inmi.it (L.F.); pietro.spezia@inmi.it (P.G.S.); fabrizio.carletti@inmi.it (F.C.); fabrizio.maggi@inmi.it (F.M.); 2 Bioinformatics Research Unit in Infectious Diseases, National Institute for Infectious Diseases Lazzaro Spallanzani—IRCCS, 00149 Rome, Italy; 3 Department for Innovation in Biological Agro-Food and Forest Systems, University of Tuscia, 01100 Viterbo, Italy; adeel.abbasi@unitus.it (A.M.A.); daniele.pietrucci@unitus.it (D.P.); n.sanna@unitus.it (N.S.); 4 Laboratory of Applied and Environmental Microbiology, Department of Chemistry, Biology and Biotechnology, University of Perugia, 06123 Perugia, Italy; maya.petricciuolo@unipg.it (M.P.); agnese.carnevali@dottorandi.unipg.it (A.C.); ermanno.federici@unipg.it (E.F.); 5 CNR-ISTP (Istituto per la Scienza e Tecnologia Dei Plasmi), 70126 Bari, Italy; 6 Dompé Farmaceutici SpA, EXSCALATE, 80131 Napoli, Italy; carmine.talarico@dompe.com; 7 Department of Experimental Medicine, University of Rome “Tor Vergata”, 00133 Rome, Italy

**Keywords:** wastewater-based epidemiology, metagenomic shotgun sequencing, virome, public health surveillance, *Anelloviridae*, Kraken2

## Abstract

Wastewater-based surveillance has emerged as a powerful approach for population-level monitoring of pathogen circulation in a timely and non-invasive manner. In this study, shotgun metagenomic sequencing was applied to wastewater samples collected from a hospital (HP), a university campus (UN), and a wastewater treatment plant (WTP). Viral sequences were taxonomically classified using Kraken2. Specifically, HP samples showed the highest viral richness, followed by WTP and UN samples (HP vs. UN, *p* = 0.0003; WTP vs. UN, *p* = 0.0018). Using Jaccard distance, significant differences were observed between WTP and UN (R^2^ = 0.181, *p* < 0.001), WTP and HP (R^2^ = 0.159, *p* < 0.001), and UN and HP (R^2^ = 0.223, *p* < 0.001), and similarly, for Sørensen–Dice dissimilarity: WTP vs. UN (R^2^ = 0.238, *p* < 0.001), WTP vs. HP (R^2^ = 0.212, *p* < 0.001), and UN vs. HP (R^2^ = 0.307, *p* < 0.001). Human-associated viral families were detected across all sources, predominantly *Poxviridae*, *Orthoherpesviridae*, *Polyomaviridae* and *Circoviridae*. Furthermore, the taxonomic composition of indirectly associated viruses, mainly *Anelloviridae* and *Crassvirales*, was examined. Overall, these findings support the potential of wastewater metagenomics as a reliable tool for monitoring viral diversity within environmental and public health contexts, although further research is needed to establish its operational utility for routine surveillance applications within a One Health framework.

## 1. Introduction

Wastewater-based epidemiology (WBE) has emerged as a powerful approach for monitoring pathogenic organisms at the population level [1]. Wastewater represents a dynamic, information-rich matrix that captures biological, chemical, and microbial signatures derived from human activity, integrating faecal, urinary, and respiratory secretions along with associated microbial communities, environmental contaminants, and residual pharmaceuticals. This multifaceted composition offers an unparalleled opportunity to infer population-level health dynamics through the analysis of a single, composite sample. Over the past decade, WBE has emerged as a transformative approach for public health surveillance, providing a non-invasive, cost-effective, and real-time tool to monitor community-level circulation of infectious agents and other health-related biomarkers [2,3,4].

Historically, the practice of detecting viral pathogens in wastewater dates back to the 1960s, when environmental surveillance of poliovirus played a pivotal role in global poliomyelitis eradication campaigns. These pioneering efforts laid the conceptual foundation of WBE, later expanded to include the detection of pharmaceutical residues and illicit drugs in the late 1990s and early 2000s. Over the past two decades, WBE has evolved into a multidisciplinary surveillance paradigm encompassing not only infectious disease monitoring but also the tracking of antimicrobial resistance, pharmaceutical pollution and environmental contaminants [5,6,7].

The COVID-19 pandemic further underscored the relevance of WBE as an early warning system for emerging infectious diseases. By detecting viral RNA in sewage before clinical cases were reported, wastewater monitoring provided critical temporal and spatial insights into SARS-CoV-2 transmission dynamics, including among asymptomatic populations [8,9,10]. Moreover, the integration of WBE data with AI-driven predictive modelling and global data-sharing platforms such as the WHO-WBE network has enabled the development of early outbreak forecasting frameworks [11].

Beyond the targeted detection of specific pathogens, the advent of metagenomic shotgun sequencing (MSS) has revolutionised the field, transforming wastewater surveillance from a pathogen-specific diagnostic tool into a comprehensive, untargeted, and hypothesis-free approach [12].

MSS is an untargeted approach that allows for the simultaneous recovery of all genetic material present in wastewater samples, providing a view of the urban microbiome and virome. Unlike amplicon-based methods, which are limited to predefined genomic regions, MSS captures both known and novel viral genomes, providing unprecedented resolution for exploring viral diversity and evolutionary dynamics in environmental matrices [13]. In wastewater viromics, MSS enables the identification of RNA and DNA viruses from multiple host sources and the reconstruction of nearly complete viral genomes, facilitating molecular epidemiology and variant tracking [14,15]. However, this comprehensive coverage poses analytical challenges, as viral reads represent a small fraction of the total metagenomic data [16]. Accurate identification, therefore, relies on optimised viral enrichment protocols and curated bioinformatics pipelines capable of distinguishing true viral sequences from bacterial or host-derived contaminants. As sequencing costs continue to decline, metagenomic approaches are expected to become a mainstay of wastewater-based surveillance, enabling real-time monitoring of viral evolution and the early detection of emerging pathogens as part of a One Health approach [17].

Metagenomic sequencing enables the simultaneous detection of thousands of microorganisms, including bacteria, viruses, fungi, and protozoa, as well as mobile genetic elements, antimicrobial resistance (AMR) genes, and other genetic markers of public health relevance [18,19]. This approach enables the simultaneous detection of known and novel pathogens and the characterisation of microbial community shifts associated with anthropogenic and environmental pressures, seasonal changes, and public health interventions. Recent studies have demonstrated the power of metagenomic approaches to resolve the spatio-temporal dynamics of viral communities in wastewater [20]. For example, large-scale longitudinal sequencing campaigns have revealed hundreds of human and animal viruses, uncovering correlations between viral abundance patterns in sewage and clinical incidence data for pathogens such as SARS-CoV-2, *Influenza*, and *Norovirus* [4]. Similarly, the wastewater virome has been shown to reflect population-level viral diversity, evolutionary trends and host–virus interactions, thereby serving as an indicator of both human health and environmental viral ecology [21].

In parallel, metagenomic investigations have highlighted the role of wastewater as a reservoir of antimicrobial resistance genes and virulence factors, underscoring the potential of this approach for One Health surveillance that integrates human, animal, and environmental health dimensions. Despite these advances, several challenges hinder the widespread adoption of metagenomic-based WBE [22]. These include the lack of standardised protocols for sample collection, nucleic acid extraction, and bioinformatic analysis; difficulties in quantitative normalisation across heterogeneous samples; and the need to translate complex genomic data into actionable public health insights [23]. Addressing these gaps is essential to unlock the full predictive and preventive potential of wastewater metagenomics.

Despite the growing number of wastewater metagenomic studies in recent years, significant knowledge gaps remain. Most surveillance programs, also in Italy, have focused on the targeted identification of specific pathogens, particularly SARS-CoV-2 [24,25], while relatively few studies have characterised the broader viral communities circulating in wastewater using untargeted shotgun metagenomics [4,26,27,28,29]. While most of these studies have primarily examined influents from urban wastewater treatment plants, relatively little is known about how viral community composition differs among wastewater sources representing distinct population groups, including healthcare facilities or university campuses [27,28]. Comparative datasets generated from these settings remain limited, hampering our understanding of how demographic, behavioural, and health factors influence wastewater viromes. Addressing these gaps is essential to assess the added value of site-specific wastewater surveillance and to identify viral signatures that may be associated with population contexts.

Our research group has previously demonstrated the effectiveness of wastewater genomic surveillance for tracking SARS-CoV-2 subvariants evolution, highlighting the ability of wastewater sequencing data to detect viral lineages earlier than clinical testing [30]. Building on this experience, the present study extends this analytical framework, applying high-throughput MSS to wastewater samples collected from three distinct sources: Hospital (HP), University Campus (UN) and Wastewater Treatment Plant (WTP). These sampling contexts offer complementary perspectives on pathogen circulation. Hospital effluents concentrate clinically relevant pathogens and antimicrobial resistance determinants, reflecting direct inputs from hospitalised patients and healthcare settings. In contrast, University sewage captures signals from a younger, highly mobile population, potentially characterised by different viral exposure and transmission patterns. Finally, influent from municipal wastewater treatment plants integrates inputs from diverse residential and commercial areas, offering a whole community-scale representation of pathogen circulation.

The specific aims of this study were to: (i) characterise the structure and diversity of viral communities across wastewaters representing different populations, (ii) identify human-related viral taxa, and (iii) evaluate the potential of metagenomic wastewater analysis as a routine component of public health surveillance systems. To the best of our knowledge, this is the first study in Europe to use untargeted metagenomic virome analysis to directly compare wastewater collected from a hospital, a university campus, and a municipal wastewater treatment plant.

By integrating viromic and ecological insights, this research contributes to the growing evidence base supporting wastewater metagenomics as a scalable, real-time and holistic platform for monitoring pathogen circulation, antimicrobial resistance dissemination and community health dynamics.

## 2. Results

### 2.1. Taxonomic Richness Across Sampling Sites

**Figure 1:**
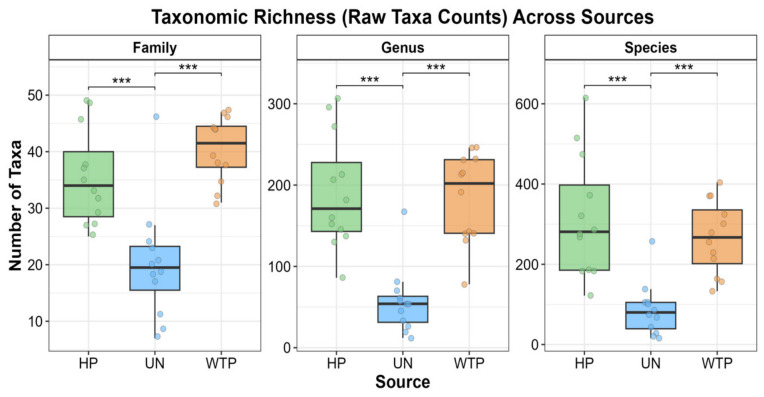
Taxonomic richness across different ranks and sources based on raw abundance. The “***” annotations represent the strength of statistical difference between comparison groups.

The number of taxa was detected per sample, grouped by sampling site and amplification technique, shown at three (family, genus and species) taxonomic ranks (Figure 1). Boxplots illustrate the variability in viral diversity, from raw taxa counts, across samples from the HP, WTP and UN (*p* < 0.001). Overall, HP and WTP samples exhibited higher taxonomic richness across all ranks, whereas UN samples consistently showed a lower number of detected taxa. This trend was observed for family, genus and species-level classifications, indicating differences in viral community complexity across sampling environments, particularly reduced complexity in UN samples.

### 2.2. Diversity Analysis

**Figure 2:**
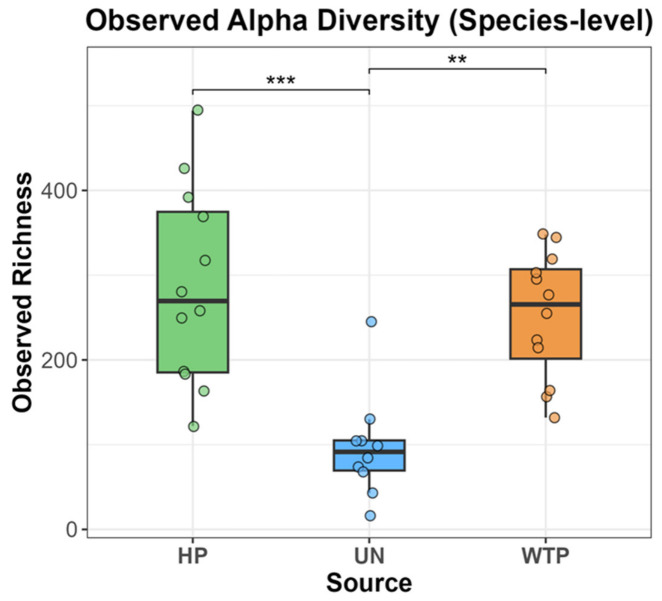
Observed richness at the species level across sources based on rarefied data. The “**/***” annotations represent the strength of statistical difference between comparison groups.

The Kruskal–Wallis test indicated significant differences in alpha diversity metrics (Figure 2) across sources for Observed species richness (*p* = 0.0001, ***); specifically, HP samples had the highest richness, followed by WTP and UN samples (mean HP = 287, WTP = 252, UN = 97). Dunn’s post hoc comparisons revealed HP and WTP had significantly higher richness than UN samples (HP vs. UN, *p* = 0.0003; WTP vs. UN, *p* = 0.0018).

**Figure 3:**
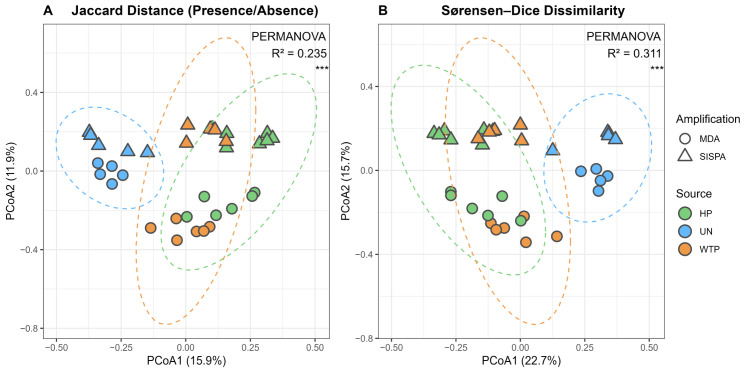
Beta diversity at the species level; PCoA (adonis2, distance~Source) based on Jaccard distance (**A**) and Sørensen–Dice dissimilarity (**B**). Samples are coloured by source (HP, UN, WTP) and shaped by amplification method for visual representation only (Multiple Displacement Amplification (MDA): circles; Sequence-Independent Single-Primer Amplification (SISPA): triangles). Dashed ellipses indicate group dispersion. Percentages on axes represent explained variance. PERMANOVA shows a significant effect of source (Jaccard: R^2^ = 0.235; Sørensen–Dice: R^2^ = 0.311; *p* = 0.0001). The “***” annotations represent the strength of statistical difference between comparison groups.

Beta diversity analyses were conducted to compare the viral community composition across three sources using two distance metrics, Jaccard distance and Sørensen–Dice Dissimilarity (Figure 3). The results revealed a clear separation of microbial communities according to sample source. Global PERMANOVA indicated that source explained a significant proportion of the observed variation using both Jaccard distance (R^2^ = 0.235, *p* < 0.001, Figure 3A) and Sørensen–Dice dissimilarity (R^2^ = 0.311, *p* < 0.001, Figure 3B), demonstrating a robust effect across distance metrics. Pairwise comparisons confirmed significant differences between all group pairs. Using Jaccard distance, significant differences were observed between WTP and UN (R^2^ = 0.181, *p* < 0.001), WTP and HP (R^2^ = 0.159, *p* < 0.001), and UN and HP (R^2^ = 0.223, *p* < 0.001). Similarly, Sørensen–Dice dissimilarity yielded significant results for all comparisons, with consistently higher R^2^ values: WTP vs. UN (R^2^ = 0.238, *p* < 0.001), WTP vs. HP (R^2^ = 0.212, *p* < 0.001), and UN vs. HP (R^2^ = 0.307, *p* < 0.001). Overall, the greatest divergence was observed between UN and HP samples, while WTP showed an intermediate level of dissimilarity relative to the other groups. The Sørensen–Dice metric provided greater discriminatory power than Jaccard, as reflected by the consistently higher R^2^ values.

To account for the potential effect of the amplification method, we performed an additional two-factor PERMANOVA including both source and amplification method as explanatory variables. Source remained the primary driver of community structure (Jaccard R^2^ = 0.235; Sørensen–Dice R^2^ = 0.311, both *p* < 0.0001), whereas amplification method explained a smaller, although significant, proportion of the variance (Jaccard R^2^ = 0.103; Sørensen–Dice R^2^ = 0.138, both *p* < 0.0001). These results indicate that the observed differences in community composition are primarily associated with sample source rather than with the amplification protocol.

### 2.3. Metagenomic Detection and Abundance of Human-Related Viruses

**Figure 4:**
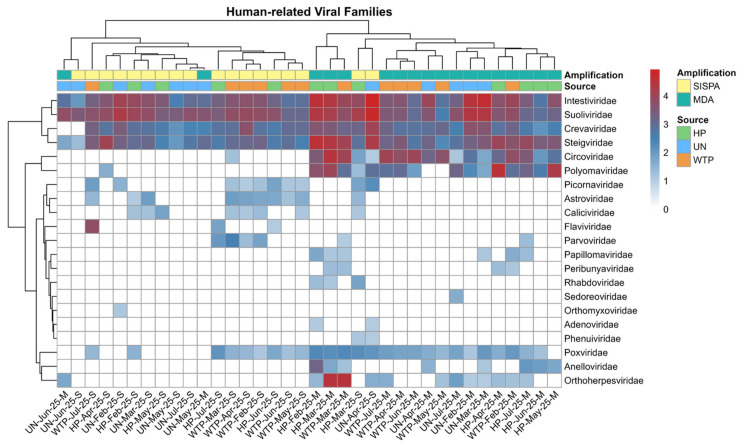
Human-related viral families detected across sources. Hierarchical clustering of log-transformed abundance data illustrates relationships among viral families (rows) and samples (columns).

**Figure 5:**
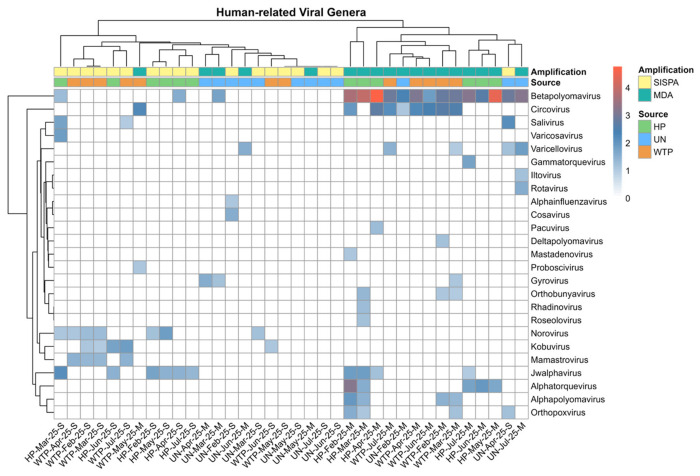
Human-related viral genera detected across sources. Hierarchical clustering of log-transformed abundance data illustrates relationships among viral genera (rows) and samples (columns).

The viral families and their respective genera related to human health were selected to further summarise as human-related viruses in this study. Specifically, Figure 4 and Figure 5 represent hierarchical clustering for selected viral families and genera across samples collected between February and July 2025 and processed using both amplification methods. Among the viral families analysed, *Poxviridae*, *Orthoherpesviridae* and *Polyomaviridae* showed higher abundance across all sample types and amplification methods, indicating their widespread presence in HP, UN and WTP. These families consist of double-stranded DNA (dsDNA) viruses, which are predominantly detected through MDA amplification, known for its preferential amplification of DNA genomes. Their consistent detection suggested stable circulation or environmental persistence of these dsDNA viruses. In contrast, *Circoviridae* and *Anelloviridae* are single-stranded DNA (ssDNA) viruses. Despite their differing genome structure compared to dsDNA viruses, they were also primarily detected through MDA, reflecting the method’s capacity to amplify DNA viral genomes. Both DNA viruses, also show enhanced detection with MDA, predominantly in HP and WTP samples. Conversely, *Picornaviridae* and *Astroviridae* are RNA virus families and were exclusively detected using SISPA amplification, which is more suitable for RNA viral genomes.

Among the dsDNA virus families, genera such as Orthopoxvirus and *Alphapolyomavirus* were detected primarily during the earlier months of sampling in HP and WTP samples, whereas *Betapolyomavirus* was consistently detected across most samples, particularly when amplified using MDA. Consistent with these observations, the ssDNA genera *Alphatorquevirus*, *Gyrovirus* and *Gammatorquevirus* were more frequently identified in samples processed with MDA, confirming the preferential amplification of DNA viruses by this method. In contrast, RNA virus genera including *Mamastrovirus*, *Norovirus* and *Kobuvirus* were detected almost exclusively in SISPA-processed samples across all three sample sources, albeit at different time points, reflecting both the RNA genome nature and the amplification specificity of SISPA. Additionally, a subset of genera was observed at only a single time point, including *Alphainfluenzavirus*, *Cosavirus*, *Rotavirus* and *Mastadenovirus*, suggesting sporadic or transient occurrence in the sampled environments.

After an overall assessment of viral diversity across the three sampling sites (UN, HP and WTP), we investigated more about some viruses (e.g., *Anelloviridae* Family and *Crassvirales* Order). Although *Crassvirales* are bacteriophages rather than human-infecting viruses, they were included in this analysis due to their known association with the human gut microbiome and their proposed role as indicators of human faecal input in environmental samples.

### 2.4. Human-Associated Viral Indicators: Anelloviridae and Crassvirales

#### 2.4.1. Characterisation of *Anelloviridae* Family

**Figure 6:**
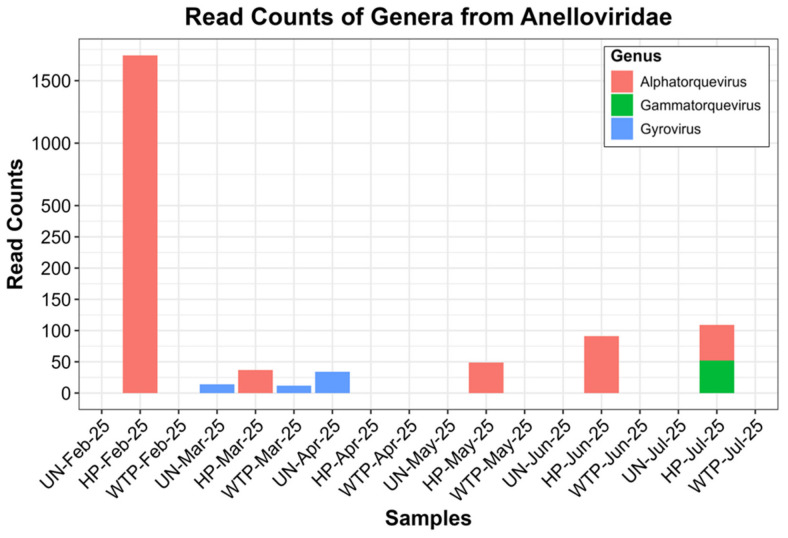
Number of raw read counts of Genera from *Anelloviridae* across samples collected from different sources and months.

We found genera of the *Anelloviridae* family (*Alphatorquevirus*, *Gammatorquevirus* and *Gyrovirus*) across samples collected monthly from HP, UN and WTP sites between February and July 2025. These genera were found in a total of eight samples (Figure 6). *Alphatorquevirus* was consistently detected in HP samples across the sampling months, with a notable peak over 1500 reads in the sample collected in February 2025, while *Gammatorquevirus* was detected only once in July in the HP sample. In contrast, *Gyrovirus* was detected in UN samples collected in March and April and in one WTP sample collected in March.

**Figure 7:**
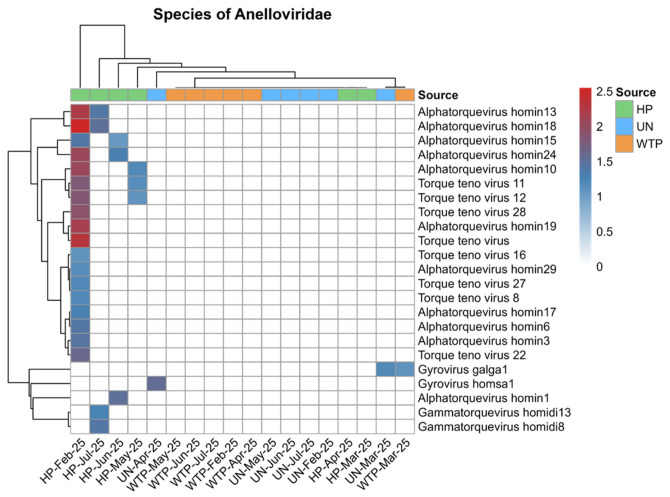
*Anelloviridae* species abundance (log-transformed) across sources.

The investigation on the classification of species of *Alphatorquevirus*, *Gammatorquevirus* and *Gyrovirus* (Figure 7) showed that 19 species were associated with the *Alphatorquevirus* genus, while only two species were associated with the *Gammatorquevirus* and *Gyrovirus* genera. Notably, most species of *Alphatorquevirus* genus were detected in the HP sample collected in February. All *Anelloviruses* species detected in May, June and July in HP samples were not shared (i.e., different species are observed in different months). Overall, *Gyrovirus* species were detected only in two UN samples (March and April) and in one sample of WTP (March).

#### 2.4.2. Distribution of *Crassvirales* Across Wastewater Samples

For the *Crassvirales*, we detected four families: *Crevaviridae*, *Intestiviridae*, *Steigviridae* and *Suoliviridae*.

**Figure 8:**
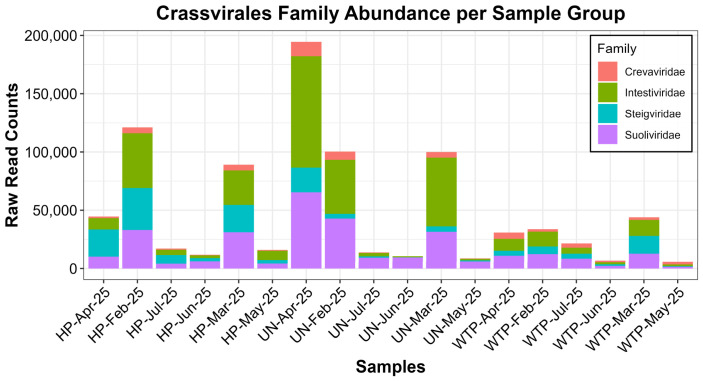
Abundance of *Crassvirales* families across sources based on raw read counts.

Figure 8 shows raw read counts of these families across three groups (UN, HP, WTP) over multiple sampling time points between February and July 2025. Comparison among groups showed that UN and HP groups generally had the highest overall abundance of *Crassvirales* families, especially evident in the peak observed in the UN sample of April 2025, where the *Intestiviridae* and *Suoliviridae* families dominated the viral community. The WTP group consistently showed the lowest raw read counts across all families and time points.

**Figure 9:**
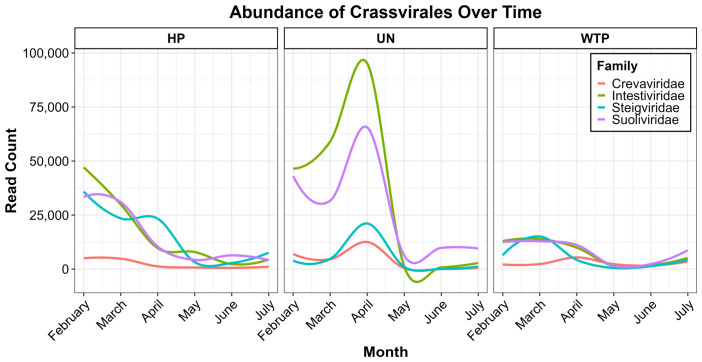
Temporal changes in the abundance of *Crassvirales* families across sources, based on raw read counts.

Within each group, temporal trends were apparent; for example, in the UN group, raw read counts increased from February to April 2025, primarily driven by *Intestiviridae* and *Suoliviridae*, then sharply decreased in the following months (Figure 9). The HP group showed a gradual decline in abundance over time across all families. The WTP group maintained relatively stable but low abundance values throughout the sampling period. Among the families, *Intestiviridae* and *Suoliviridae* were generally the most abundant across all groups, while *Crevaviridae* and *Steigviridae* contributed smaller but consistent proportions.

**Figure 10:**
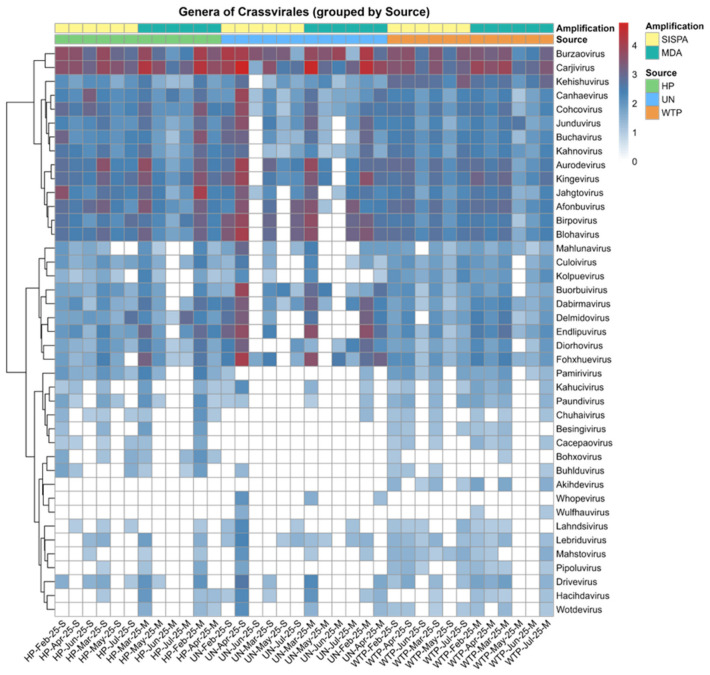
Pattern of *Crassvirales* genera grouped by source (columns) and clustered by genus (rows) based on log-transformed abundance.

Furthermore, the genera belonging to these *Crassvirales* families were investigated similarly by hierarchical clustering analysis (Figure 10). Between-group comparison revealed that the UN group exhibited lower genus-level richness compared to the other two sources.

The HP group showed an intermediate pattern, with many genera persistently present but a few genera fluctuating or absent at certain time points (e.g., *Paundivirus* absent in June and July).

Within each group, temporal dynamics are evident; for example, in the UN group, genera such as *Chuhaivirus* and *Pamirivirus* are present only in an earlier month (February). Interestingly, *Whopevirus* was found only in UN samples and only in earlier months (March and April). The WTP and HP groups show fewer fluctuations, implying more consistent *Crassvirales* populations across sampling time points. Some genera, including *Burzaovirus* and *Carjivirus*, remain constantly present in all groups and time points, representing core and consistent members of the *Crassvirales* community (100% of prevalence).

## 3. Discussion

This study applied MSS to characterise viral communities in wastewater collected from three distinct environments (HP, UN and WTP) over a six-month period. By integrating taxonomic profiling, diversity analyses and targeted investigation of human-associated viral taxa, we evaluated the potential of metagenomic wastewater surveillance as a routine public health monitoring tool. Our findings demonstrate clear site-specific differences in viral richness, diversity and community composition, highlighting the influence of population structure and environmental context on wastewater viromes. Alpha diversity analyses consistently showed that HP and WTP samples harboured significantly greater viral richness compared to UN samples. Observed richness was markedly higher in HP and WTP. The lower viral species richness observed in university samples compared with hospital and wastewater treatment plant samples is consistent with the expected ecological characteristics of these environments. Hospitals are characterised by a continuous influx of individuals with diverse clinical conditions and microbial exposures, which may contribute to a broader spectrum of viral taxa. Similarly, wastewater treatment plants receive and concentrate input from large human populations and multiple environmental sources, resulting in highly diverse viral communities. University settings are predominantly occupied by young adults, representing a relatively homogeneous population.

Several environmental and demographic factors may contribute to the observed patterns of viral diversity across sampling sites. From an environmental perspective, differences in human occupancy, frequency of use, cleaning and disinfection practices, ventilation, and the persistence of viral particles on surfaces may influence viral community composition.

From a demographic perspective, the populations associated with different environments are likely to vary in age distribution, health status, and overall heterogeneity. University environments are predominantly occupied by young adults and therefore represent a relatively homogeneous population. In contrast, hospitals are frequented by individuals with diverse clinical conditions and age groups, while wastewater treatment plants integrate contributions from large and heterogeneous communities. Such differences may increase the range of viral taxa introduced into these environments and could partly explain the higher viral richness observed in hospital and wastewater samples compared with university samples.

Together, these findings reinforced the concept that wastewater viromes mirror the characteristics of contributing populations, highlighting site-specific differences in microbial community richness and diversity. Beta diversity analyses further supported this interpretation, as results revealed significant differences in viral community composition among all source types, with the greatest separation observed between HP and UN samples. The parallel application of SISPA and MDA provided important methodological insights, as MDA preferentially enriched DNA viruses, particularly double-stranded DNA families such as *Poxviridae*, *Orthoherpesviridae* and *Polyomaviridae*, whereas SISPA facilitated the detection of RNA viruses including *Picornaviridae* and *Astroviridae*. These results highlight both the strengths and inherent biases of amplification-based metagenomic workflows, demonstrating that methodological choices significantly shape the observed virome composition.

In particular, SISPA and MDA are known to introduce amplification biases that may preferentially enrich specific viral genome types while underrepresenting others, thereby affecting both relative abundance estimates and diversity of metrics. Consequently, differences observed among viral taxa should be interpreted cautiously, as they may reflect a combination of biological variation and methodological effects. Despite these limitations, the detection of a broad range of viral families across multiple wastewater sources demonstrates the utility of metagenomic approaches for characterising viral community composition and exploring patterns of virome diversity.

These findings further highlight the value of wastewater as a surveillance tool capable of capturing viral diversity beyond that observed in clinical settings. This is not surprising, as wastewater-based virology often reflects a broader, less clinically influenced picture of viral circulation within a population.

Within this broader viral landscape, the widespread detection of *Anelloviridae* and *Polyomaviridae* across the investigated wastewater samples likely reflects the high prevalence of these viruses in the contributing human populations. Because both viral families are commonly associated with persistent or chronic infections and can be shed by asymptomatic individuals, their occurrence in wastewater is consistent with the continuous input of human-derived viral material into sewer systems. In this context, *Anelloviridae* may serve as indicators of the overall human virome represented within the catchment population, while their abundance has also been proposed as a proxy of population-level immune status [31,32].

Similarly, the detection of *Polyomaviridae* across multiple sites is consistent with the widespread circulation and lifelong persistence of human polyomaviruses in the general population. Rather than indicating localised disease outbreaks, their presence in wastewater likely reflects the cumulative contribution of infected individuals within the serviced communities. The detection of these viral families therefore highlights the ability of wastewater metagenomics to capture population-scale viral signatures that are not necessarily represented in clinical surveillance data, providing a more comprehensive picture of community viral dynamics [33,34,35]. Overall, *Polyomaviridae* are important opportunistic pathogens whose clinical relevance lies in their ability to persist in the host and cause severe disease when immune control is compromised. Conversely, RNA viruses including *Norovirus*, *Kobuvirus* and *Mamastrovirus* were primarily identified in SISPA-processed samples, consistent with genome type and amplification specificity. These viruses were included in the analysis due to their known association with human gastroenteritis. Noroviruses are highly infectious and a major cause of outbreaks, whereas *Kobuviruses* and *Mamastroviruses* are linked to milder gastroenteritis, particularly in children and immunocompromised individuals [36,37]. Their detection in wastewater provides insights into population-level circulation of enteric viruses [38]. A focused analysis of the *Crassvirales* order revealed marked presence in all sources, with WTP samples exhibiting relatively stable genus-level richness, UN samples showed temporal fluctuations, including an April peak dominated by *Intestiviridae* and *Suoliviridae* and HP samples displaying an intermediate pattern with a gradual decline in abundance over time. The consistent presence across all groups of *Intestiviridae* supported their role as part of a conserved core virome [39]. As *Crassvirales* bacteriophages are known to infect gut-associated bacteria and are considered indicators of human faecal input and gut microbial ecology, their relative stability in municipal wastewater likely reflected continuous and heterogeneous faecal contributions from a large population base, whereas fluctuations in the University setting may correspond to academic calendar dynamics, population mobility, or short-term epidemiological events. *Crassvirales* are not considered pathogenic to humans and represent a major component of the human gut virome. Recent studies have highlighted their potential ecological importance within gut microbial communities, although their functional role remains incompletely understood [40].

The viral diversity observed in this study is consistent with previous metagenomic investigations of wastewater viromes conducted across geographic and urban settings. Building-level surveillance studies have demonstrated that wastewater viromes reflect the characteristics of the contributing populations, with university wastewater enriched in human polyomaviruses such as JC and BK viruses, while other settings, including nursing homes, exhibit distinct viral signatures characterised by differing proportions of *Astroviridae*, *Picornaviridae*, *Polyomaviridae* and *Papillomaviridae* [27]. Similar patterns have been reported in municipal wastewater systems, where human-associated viral families including *Polyomaviridae*, *Papillomaviridae*, *Parvoviridae*, *Astroviridae*, and *Picornaviridae* are consistently detected across diverse populations [41]. Moreover, recent large-scale international surveys have identified a conserved core wastewater virome shared among geographically distant cities, despite substantial local variation in viral composition [42].

These findings further highlight the value of wastewater surveillance as a complementary approach to clinical monitoring, providing a more comprehensive representation of community-level viral diversity that includes both symptomatic and asymptomatic infections.

Our study has some limitations that likely affected both sensitivity and interpretation. First, the use of one composite sample per site per month with limited temporal resolution and may have missed short-lived fluctuations or low-abundance viral signals, which are known to vary across time and location in wastewater viromes [4,43]. Although composite sampling can be considered more representative than a single grab sample, it does not eliminate biases related to low viral concentrations and may introduce degradation during collection and storage [43,44]. Second, sequencing reads should not be interpreted as a direct measure of viral load, because observed abundance is influenced by wastewater hydrology, sample processing, enrichment strategy, sequencing depth and differential recovery across viral targets [45,46]. Accordingly, metagenomic data in this study are better interpreted as a qualitative or semi-quantitative overview of the detectable virome rather than as absolute measures of community infection burden [13,16]. Third, because we did not evaluate associations with population density, seasonality or physicochemical wastewater characteristics, we cannot determine whether between-site differences reflect true epidemiological variation or matrix-driven effects [44,47]. Finally, taxonomic assignments, especially for low-abundance or potentially novel viruses, should be interpreted cautiously, as short-read metagenomics in complex wastewater matrices can be affected by incomplete databases, mapping ambiguity and limited genome coverage. For this reason, the identification of specific viruses has sometimes been achieved with additional analyses to improve confidence, such as contig assembly, phylogenetic analysis, replicate strategies and targeted molecular validation [41,43,47].

## 4. Materials and Methods

### 4.1. Experimental Design

**Figure 11:**
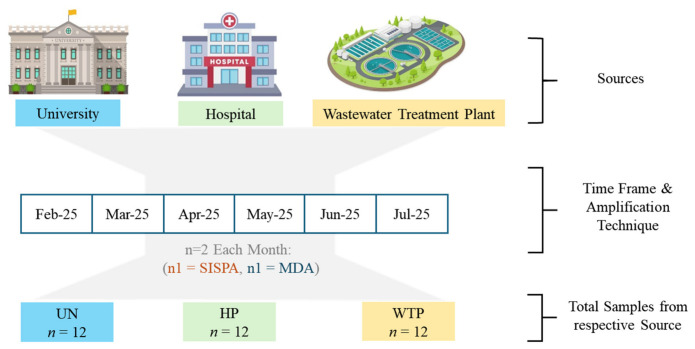
Experimental Design and Wastewater Shotgun Metagenome Samples. Each source (University, Hospital, Wastewater Treatment Plant) resulted in 12 (*n* = 6 MDA, *n* = 6 SISPA) samples over the span of 6 months.

Wastewater samples were collected monthly from February 2025 to July 2025 at three distinct sites, namely HP, UN and WTP, all located in the city of Perugia, Italy within a maximum distance between each other of 6 km. Importantly, the WTP did not receive hospital sewage. At each site and each monthly time point, one composite sample was collected by pooling together three grab sub-samples of 1 L each, yielding a total of 18 primary samples across the study period (3 sites × 6 months). Each sample was amplified using two separate amplification methods during library preparation, Sequence-Independent Single-Primer Amplification (SISPA) and Multiple Displacement Amplification (MDA) for RNA and DNA-based viruses, respectively. Both methods were applied to every primary sample, resulting in a total of 36 shotgun-sequenced datasets derived from 18 independent biological samples (Figure 11).

### 4.2. Viral Nucleic Acid Extraction

Viral nucleic acid extraction was carried out for each collected sewage sample following a validated protocol [48], as previously described [30]. Briefly, 100 mL of each sample was centrifugated at 4500× *g* for 30 min at 4 °C to remove debris and minimise bacterial contamination. Then, PEG8000-NaCl concentration was performed to enrich for viral particles present in the sample. Finally, nucleic acid extraction was carried out with a magnetic bead-based procedure using the eGENE-UP system (bioMerieux, Marcy-l’Étoile, France), following the manufacturer’s instructions. TE-eluted RNA samples were purified with the OneStep PCR Inhibitor Removal Kit (Zymo Research, Irvine, CA, USA), supplemented with one U/μL of RiboLock RNase Inhibitor (Thermo Fisher Scientific, Waltham, MA, USA) and stored at −80 °C.

### 4.3. Library Preparation and Shotgun Sequencing

Extracted samples were then processed by two different approaches, with the aim of enriching both RNA and DNA components. The SISPA technique was used to enrich the RNA component [49] without performing a DNAse pretreatment in order to prevent DNA fragment loss, while the MDA method enriches the DNA component and is particularly suitable for increasing the probability of detecting viral circular genomes. The SISPA approach is based on a random reverse transcription performed with a tagged primer, which is then used for subsequent amplification of the cDNA products. MDA is based on the use of the Phi29 enzyme to obtain the PCR product by random primers and isothermal amplification. SISPA and MDA obtained amplicons that were then used for library preparation, starting from 100 ng of DNA. The library was prepared using the Ion Xpress Plus Fragment Library kit (ThermoFisher, Waltham, MA, USA), and sequencing was performed on the Gene Studio S5 Prime Platform (GSS5) with 540/550 chips (ThermoFisher, Waltham, MA, USA), obtaining on average 50 × 10^6^ reads per sample.

### 4.4. Data Processing and Bioinformatics Analysis

Ion Torrent raw data bam files were converted to FASTQ format using samtools software (v.1.13) [50], and poor or low-quality reads and adapters with an average quality score below 30 in the raw data were trimmed and filtered using Fastp (v.0.23.4) [51].

Quality-filtered raw sequences were subsequently subjected to taxonomic classification using the Kraken2 v.2.1.3 classification system https://github.com/DerrickWood/kraken2 (accessed on 9 December 2025), a k-mer-based taxonomic assignment tool that identifies the most likely taxon for each read by matching exact k-mer sequences against a reference database and applying the Lowest Common Ancestor (LCA) algorithm to resolve ambiguous hits [52]. A comprehensive Kraken2 database (Standard PlusPFP database release of December 2024), https://benlangmead.github.io/aws-indexes/k2 (accessed on 23 December 2025) was used, containing genomic sequences from bacteria, viruses, archaea, fungi, plants, plasmid and protozoa. This approach computationally enabled rapid assignment of reads to a taxonomic category (species, genus or higher-level taxa) [53]. Kraken2 was executed using default confidence score and parameters (k  =  35, ℓ  =  31, s = 7). To obtain accurate estimates of taxon abundances, the Bracken v.3.0.1 (Bayesian Reestimation of Abundance with Kraken), https://github.com/jenniferlu717/Bracken (accessed on 9 December 2025) tool was subsequently used [54]. Bracken refines the initial Kraken2 classifications by re-estimating the number of reads originating from each taxon, based on the distribution of k-mer assignments across different taxonomic levels. This approach provides more reliable estimates of relative abundance at various ranks (order, family, genus, and species).

Subsequently, KrakenTools v.1.2.1, a suite of Python-based utilities designed to process and analyse Kraken2 output files, was used to extract reads classified as viral https://github.com/jenniferlu717/KrakenTools (accessed on 9 December 2025). This step enabled downstream analyses focused specifically on the viral component of the dataset.

To prioritise viruses with potential relevance to human health, we focused on viral families associated with human infections. A reference list was obtained from the “Human Viruses” section of ViralZone https://viralzone.expasy.org/ (accessed on 11 February 2026) [55], which was used exclusively as a metadata resource to retrieve human-associated viral taxa. This list was subsequently used to screen the dataset, and patterns were visualised through hierarchical clustering analysis. In particular, we conducted a targeted downstream analysis of viral taxa, by hierarchical clustering, starting with members of the *Anelloviridae* family and viral families belonging to the *Crassvirales* order. The *Anelloviridae* family and its associated genera were included in the analysis due to their role as common constituents of the human virome and their proposed use as indicators of host immune status. *Crassvirales* are a highly abundant and persistent group of bacteriophages within the human gut virome, where they act as a keystone component by modulating viral community structure and indirectly influencing microbial ecosystem dynamics through their interactions with bacterial hosts.

All analyses were performed on CINECA (Interuniversity Consortium in the North-East for Automatic Computing) supercomputing servers.

### 4.5. Statistical Analysis

Statistical analyses were carried out in R Studio software (v, 2025.05.1, Build 513). To compare samples originating from different sources, data normalisation procedures were applied. The observed richness of viral communities (alpha diversity), within groups, provided insights into how diverse the taxa are in a single environment. Beta diversity, on the other hand, assessed differences in community composition between samples (between groups), highlighting how viral structures vary across sources. Both alpha and beta diversity matrices were computer based on incidence only (presence/absence) and not abundance [56].

The Kruskal–Wallis test was performed to compute alpha diversity, while the post hoc Dunn’s test with Bonferroni correction was applied when Kruskal–Wallis showed significant results. Beta diversity distance or dissimilarity was measured using the Jaccard Distance (binary) and Sørensen–Dice Dissimilarity metric followed by Principal Coordinates Analysis (PCoA) representation [57,58].

Significant differences in community composition between sources were tested using Permutational Multivariate Analysis of Variance (PERMANOVA) via the adonis2 (distance~Source) function from the vegan R package (v.2.7-2), with 99,999 permutations [59]. Pairwise PERMANOVA tests were subsequently performed to assess pair-wise differences between Sources.

Additionally, to evaluate whether the use of two amplification methods (SISPA and MDA) influenced the global PERMANOVA results, an additional two-factor PERMANOVA model was performed including both amplification method and sample source as explanatory variables (distance~Amplification_technique + Source, by = “margin”). This approach allowed the independent contribution of each factor to community dissimilarity to be assessed while controlling for the effect of the other. Analyses were performed using both Jaccard and Sørensen–Dice distance matrices with permutations, consistent with the global and pairwise PERMANOVA analyses described above.

All R packages used in R studio are listed in the Supplementary Material.

## 5. Conclusions

Collectively, these findings support the integration of shotgun metagenomics into routine wastewater-based epidemiology. The ability to differentiate site-specific virome structures suggested that tailored surveillance strategies could be developed according to monitoring objectives, for example, using HP wastewater for early detection of clinically relevant or emerging pathogens and municipal influent for population-level dynamics. Differences in richness, composition and temporal dynamics reflected the characteristics of contributing populations, reinforcing wastewater metagenomics as a scalable, real-time platform for integrated public health surveillance. Overall, this study demonstrated that metagenomic wastewater surveillance captures distinct and meaningful viral community structures across different environmental and demographic contexts.

Importantly, the approach used enabled the initial characterisation of highly heterogeneous wastewater samples, providing a valuable foundation for future investigations. However, further methodological improvements, particularly through the development of more accurate and sensitive bioinformatic pipelines, will be essential to improve taxonomic resolution and facilitate the interpretation of complex virome datasets.

Therefore, with continued methodological standardisation and integration with clinical datasets, this approach holds strong potential to contribute to early warning systems and to monitoring of community health dynamics within a One Health framework.

## Data Availability

The datasets generated and analysed during this study are available in the NCBI Sequence Read Archive. Raw sequencing data (FASTQ files) have been deposited under BioProject: PRJNA1457282.

## Supplementary Materials

The following supporting information can be downloaded at: https://www.mdpi.com/article/10.3390/ijms27146430/s1.
