# Supplementary material for: Comparative Analysis of Viral Communities in Hospital, University and Urban Wastewater by Shotgun Metagenomic Sequencing
Source: Int J Mol Sci. 2026 Jul 20;27(14):6430. doi: 10.3390/ijms27146430 (PMC13410366; doi:10.3390/ijms27146430)
Supplement: Supplementary file 1 [file ijms-27-06430-s001.zip › ijms-4372393-supplementary.pdf]

Comparative Analysis of Viral Communities in Hospital, University and Urban Wastewater by Shotgun Metagenomic Sequencing

Alessandra Nappo, Adeel Mumtaz Abbasi, Giulia Berno, Martina Rueca, Flavia Smoquina, Cesare Ernesto Maria Gruber, Lavinia Fabeni, Pietro Giorgio Spezia, Fabrizio Carletti, Daniele Pietrucci, Maya Petricciuolo, Agnese Carnevali, Nico Sanna, Carmine Talarico, Ermanno Federici, Giovanni Chillemi*, Fabrizio Maggi

Supplementary Material

R Studio packages used for the analysis

```
R version 4.5.2 (2025-10-31 ucrt)
Platform: x86_64-w64-mingw32/x64
Running under: Windows 11 x64 (build 26200)

Matrix products: default
  LAPACK version 3.12.1

locale:
[1] LC_COLLATE=English_American Samoa.utf8  LC_CTYPE=English_American Samoa.utf8
[3] LC_MONETARY=English_American Samoa.utf8 LC_NUMERIC=C
[5] LC_TIME=English_American Samoa.utf8

time zone: Europe/Rome
tzcode source: internal

attached base packages:
[1] grid      stats     graphics  grDevices utils     datasets  methods   base

other attached packages:
 [1] writexl_1.5.4        tictoc_1.2.1         stringr_1.6.0        scales_1.4.0
 [5] pairwiseAdonis_0.4.1 cluster_2.1.8.1      FSA_0.10.1           vegan_2.7-2
 [9] permute_0.9-8        ranacapa_0.1.0       ggpubr_0.6.2         pheatmap_1.0.13
[13] ggrepel_0.9.8        ggplot2_4.0.1        tibble_3.3.1         phyloseq_1.54.0
[17] tidyr_1.3.2          dplyr_1.1.4

loaded via a namespace (and not attached):
 [1] gtable_0.3.6        rstatix_0.7.3       rhdf5_2.54.1        Biobase_2.70.0
 [5] lattice_0.22-7      rhdf5filters_1.22.0 vctrs_0.6.5         tools_4.5.2
 [9] generics_0.1.4      biomformat_1.38.0   stats4_4.5.2        parallel_4.5.2
[13] pkgconfig_2.0.3     Matrix_1.7-4        data.table_1.18.0   RColorBrewer_1.1-3
[17] S7_0.2.1            S4Vectors_0.48.0    lifecycle_1.0.5     compiler_4.5.2
[21] farver_2.1.2        Biostrings_2.78.0   Seqinfo_1.0.0       codetools_0.2-20
[25] carData_3.0-6       Formula_1.2-5       car_3.1-3           pillar_1.11.1
[29] crayon_1.5.3        MASS_7.3-65         iterators_1.0.14    abind_1.4-8
[33] foreach_1.5.2       nlme_3.1-168        tidyselect_1.2.1    digest_0.6.39
[37] stringi_1.8.7       reshape2_1.4.5      purrr_1.2.1         splines_4.5.2
[41] ade4_1.7-23         cli_3.6.5           magrittr_2.0.4      survival_3.8-3
[45] broom_1.0.12        ape_5.8-1           withr_3.0.2         backports_1.5.0
[49] XVector_0.50.0      igraph_2.2.1        multtest_2.66.0     ggsignif_0.6.4
[53] IRanges_2.44.0      mgcv_1.9-3          rlang_1.1.7         Rcpp_1.1.1
[57] glue_1.8.0          BiocGenerics_0.56.0 rstudioapi_0.18.0   jsonlite_2.0.0
[61] R6_2.6.1            Rhdf5lib_1.32.0     plyr_1.8.9
```
